# The anatomical relationship between the mandibular first molar roots and the mandibular canal based on breed size and skull type

**DOI:** 10.3389/fvets.2022.956976

**Published:** 2022-07-28

**Authors:** Erica Greene, Aaron Rendahl, Stephanie Goldschmidt

**Affiliations:** ^1^Department of Veterinary Clinical Medicine, University of Illinois, Urbana, IL, United States; ^2^Department of Veterinary Clinical Sciences, University of Minnesota, St. Paul, MN, United States

**Keywords:** oral surgery, mandibular molar, mandibular canal, dentistry, anatomy

## Abstract

The first molar is the largest tooth in the dog mandible with roots often extending to the level of the mandibular canal (MC). The anatomical relationship between the roots and MC is variable and the normal relationship between those structures in a diverse population of dogs has not been established. The lingual location of the roots relative to the MC poses a risk for iatrogenic trauma during dentoalveolar surgery, and it is unknown if certain skull conformations are predisposed to this relationship. This study aimed to identify associations between patient weight and skull type with molar tooth root location. CT scans performed for reasons unrelated to the study were retrospectively analyzed. Subjects were sorted into one of 12 groups (*n*=16 per group) based on skull type (brachycephalic, mesaticephalic, and dolichocephalic) and weight (extra small: ≤ 6.8 kg, small: >6.8 to ≤ 13.6 kg, medium: >13.6 to ≤ 25 kg, and large >25 to ≤ 38.6 kg). The mandibular first molar roots were categorized as lingual, buccal, or dorsal relative to the MC. Lingual root location was diagnosed in 50.0% of all roots evaluated, and 64.2% of all dogs assessed had at least one root in the lingual position. The size was shown to be protective, with lingual root location being significantly less likely as size increased. The exception to this was in large brachycephalic patients, which had rates of lingual roots similar to smaller dogs. Buccal roots were rarest, diagnosed in only 9.7% of all roots, and were most common in brachycephalic patients, which had 83.8% of all buccal roots. Additional caution should be employed when removing alveolar bone during surgical extraction in dogs ≤ 13.6 kg and in large brachycephalic patients (boxers) to avoid iatrogenic trauma to the neurovascular bundle.

## Introduction

The mandibular canal (MC) houses the inferior alveolar artery, vein, and nerve. It is located in the ventral mandible and extends from the mandibular to the mental foramen (1, 2). The anatomical relationship between the tooth roots and the MC can vary. Pending root localization there is a risk of damage to the structures within the MC during surgical extraction, especially if the excess buccal alveolar bone is removed.

In a study that examined 101 canine cadaver heads with mesaticephalic skull type and unknown breed, 81.4% of mandibular first molar roots were located lingually to the MC (1). Another evaluated 103 small breed dogs (under 15 kg) and revealed that 82.7% of mandibular first molar roots were located at the lingual aspect of the MC (3). These studies suggest that lingual root location relative to the MC is the most common. However, large-scale studies investigating the anatomical relationship between these two structures in a diverse patient population have not been performed. This study aimed to analyze the relationship between mandibular first molar roots and the MC in dogs of varying skull types and weights. We hypothesized that lingual mandibular first molar roots would be relatively more common in brachycephalic dog breeds and extra small ( ≤ 6.8 kg) dogs.

## Materials and methods

Helical skull CT (Toshiba Aquilion 64 CFX, Toshiba Medical Systems, Tustin, CA, USA) and cone-beam CT (CBCT, Xoran Vet Cat, Xoran Technologies, Ann Arbor, MI, USA) scans performed on client-owned animals at the University of Minnesota between 21 October 2014 and 28 April 2021 were compiled. This study did not involve the use of animals; thus it was exempt from IACUC ethical approval. Scans were initially organized by breed and then sorted into size and skull categories.

Each breed was placed into a size category using the mean adult weight listed by the American Kennel Club (4) to avoid obesity or malnutrition of specific patients affecting size categorization. Breeds were classified as extra small (≤ 6.8 kg), small (>6.8 kg, ≤ 13.6 kg), medium (>13.6 kg, ≤ 25 kg), or large (>25 kg, ≤ 38.6 kg). Giant breeds were excluded due to the rarity of patients. Breeds were classified as having brachycephalic, mesaticephalic, or dolichocephalic skull type (5). Skull classification was made clinically based on the appearance of each breed.

Following the organization of all available CT and CBCT studies into one of the 12 size/skull categories (Table 1), 16 imaging studies from each category were randomly selected using a random number generator. Scans were then evaluated and excluded if the first mandibular molar tooth was absent, the apex of the mandibular first molar was open, or there was severe surrounding oral pathology that interfered with an evaluation of either the root or the MC. Malocclusions and absent teeth, other than the mandibular first molar tooth, did not exclude patients from this study. When a patient was excluded, another was randomly selected from the same category to replace it.

**Table 1:** Breeds evaluated after a random selection of CT scans per category.

	**Brachycephalic**	**Mesaticephalic**	**Dolichocephalic**
Extra Small	Japanese Chin (1), Shih Tzu (11), Lhasa Apso (3), Pekingese (1)	Cairn Terrier (1), Chihuahua (1), Jack Russell Terrier (4), Papillon (1), Toy Poodle (3), Yorkshire Terrier (2), Norfolk Terrier (1), Havanese (1), Pomeranian (1), Miniature Poodle (1)	Excluded from analysis
Small	Boston Terrier (5), Cavalier King Charles Spaniel (4), Pug (6), French Bulldog (1)	Cocker Spaniel (6), Miniature American Eskimo (1), West Highland White Terrier (1), Pembroke Welsh Corgi (3), Beagle (1), English Cocker Spaniel (1), Rat Terrier (1), Miniature Schnauzer (2)	Shetland Sheepdog (5), Dachshund (5), Scottish Terrier (3), Fox Terrier (2), Miniature Bull Terrier (1)
Medium	English Bulldog (7), American Staffordshire Terrier (2), American Pit Bull Terrier (1), Olde English Bulldogge (3), Staffordshire Bull Terrier (3)	Standard American Eskimo (1), Australian Cattle Dog (2), English Springer Spaniel (3), Norwegian Elkhound (1), Petit Basset Griffon Vendeen (1), Standard Schnauzer (2), Brittany Spaniel (1), Siberian Husky (1), Nova Scotia Duck Tolling Retriever (1)	Afghan Hound (1), Basset Hound (7), Standard Poodle (7), Pharaoh Hound (1)
Large	Boxer (15), Chow Chow (1)	Gordon Setter (1), Labrador Retriever (6), American White Shepherd (1), Golden Retriever (4), German Wirehaired Pointer (1), German Shorthaired Pointer (2), British Labrador Retriever (1)	Doberman Pinscher (2), German Shepherd (12), Collie (1), Greyhound (1)

In the selected imaging studies, the facial index was calculated to confirm the assigned skull type as previously described (1). Briefly, skull width was measured as the distance between the medial surfaces of the zygomatic arches at their widest point. Skull length was measured from the prosthion to the point on the palate perpendicular to the nasion. Skull width was multiplied by 100 and divided by skull length to calculate the facial index. According to Millers' Anatomy of the Dog, the mean facial index is 215, 111, and 81 for brachycephalic, mesaticephalic, and dolichocephalic skull types, respectively (5). Skulls were classified as mesaticephalic if the facial index was in the range of 96–163 (1). No published ranges are available for brachycephalic or dolichocephalic skulls. Thus, skulls with facial index >163 were classified as brachycephalic and skulls with facial index <96 were classified as dolichocephalic.

Mesaticephalic and brachycephalic dogs that did not fall in the defined facial index range were excluded and replaced with an alternative patient. However, commonly accepted dolichocephalic breeds rarely had facial index <96. Thus, it was elected to evaluate the differences in all three categories using commonly accepted dolichocephalic breeds (Table 1) despite the scans having a facial index >96. As dolichocephalic skull type could not be validated, comparisons were also made between brachycephalic (>163, *n* = 16 per weight group) and normocephalic (6) skulls. The normocephalic group was the mesaticephalic and dolichocephalic groups combined (<163, *n* = 32 per weight group).

**Figure 1:**
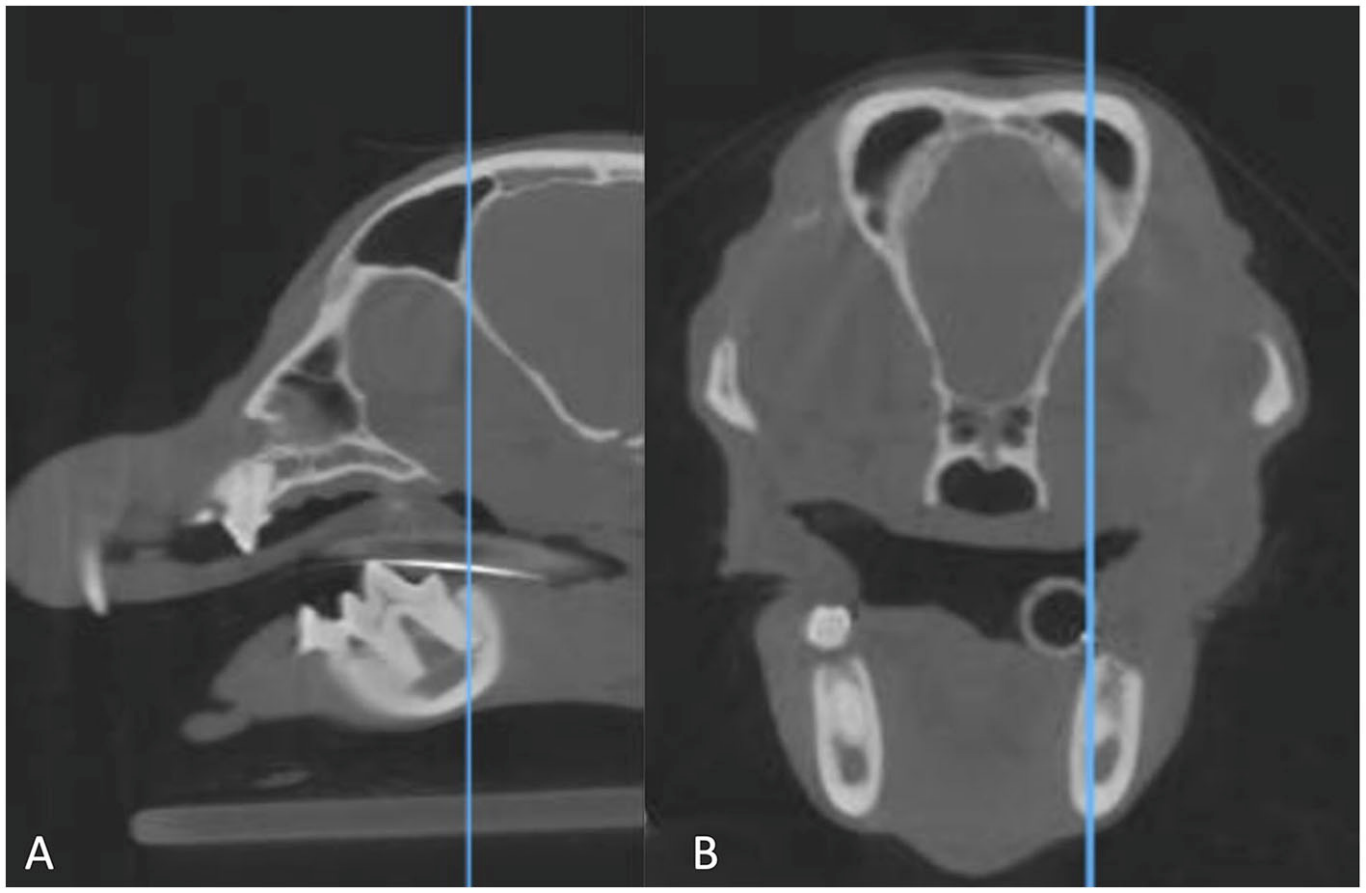
Sagittal multiplanar reformation **(A)** of a CBCT scan matched to the transverse multiplanar reformation **(B)**. The blue line in the image on the left indicates the point at which the transverse image is displayed. The line is at the longest point of the distal root of the right mandibular first molar tooth, which is where the root was evaluated.

Images were evaluated in the transverse plane where the root length was the longest as previously described (3). To facilitate analysis, the transverse image was synched with the sagittal multiplanar reformation (MPR) for simultaneous evaluation (Figure 1). The root was classified as lying lingual, buccal, or dorsal to the MC. Roots were considered dorsal if no portion of the MC was located lingually or buccally to any portion of the root (3). The mesial and distal roots of both the left and the right mandibular first molar tooth were classified separately. Additional data collected included the presence of straddle roots, defined as the mesial and distal root being on opposite sides of the MC (3), and left-right symmetry. Specifically, in regard to left-right symmetry, the position of mesial roots on the left vs. right was compared and then the position of the distal roots on the left vs. right was compared. Mesial root location was not compared to the contralateral distal root. All scans were evaluated by a single reviewer (EG).

### Statistical analysis

Power calculation performed before data collection determined that a sample size of 16 per group was needed for 80% power to detect differences of 30 percentage points between skull types or weight groups. For this calculation, data were considered to have only one tooth per dog so a multivariate logistic regression with pairwise comparisons between groups adjusted using the Tukey HSD method could be used. This overestimates the sample size, as it is conservative when compared with the actual analysis that used Kendall's test to incorporate data from all four roots for each dog.

The total number and percent of roots in each location, and of dogs with at least one root in that location, were calculated for each skull/size category individually, and for each skull type overall and each size group overall.

To test for the association of size and skull type with the number of roots in each location, Kendall's test was used, testing each location separately. For size, the size was treated as an ordinal variable, to look for an overall increase or decrease in the count with size. Each test was performed separately for each skull type as strata, and overall by using size as a block within the test. For skull type, pairwise tests were used for the three skull types with *p*-values adjusted using the Bonferroni correction, and an additional test was performed to test brachycephalic against the other two together (normocephalic). Similarly, this was performed for each size separately and overall, using skull type as a block, and for the count of straddle roots and the presence of symmetry. Due to the many ties and the small sample sizes, *p*-values were computed using simulations with 5,000 permutations. All computations was performed using R version 4.1.1 (7).

## Results

**Figure 2:**
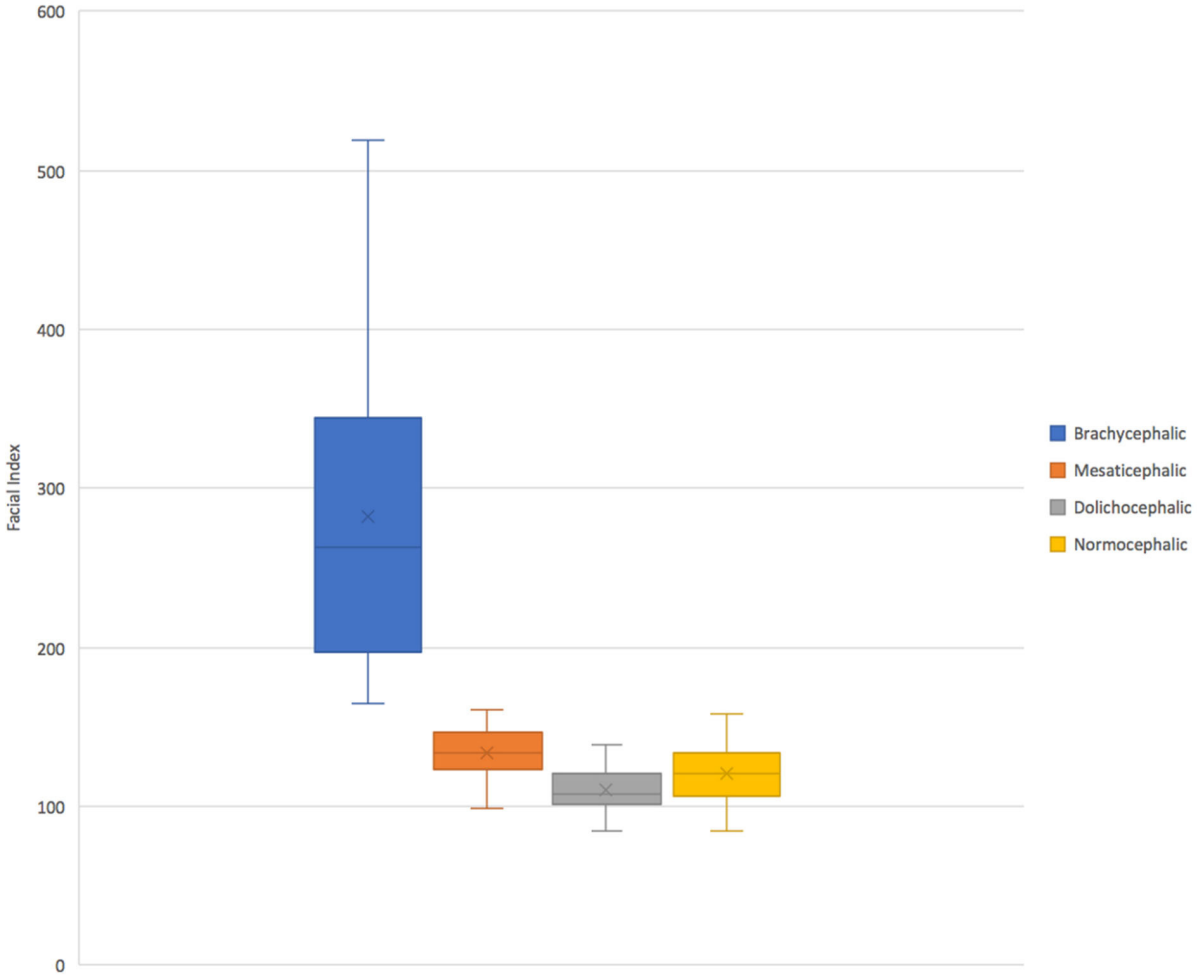
A box and whiskers plot facial index per each skull type. Normocephalic skulls were defined as all skulls with a facial index <163 and excluded extra small patients.

On retrospective analysis of the electronic medical record, 1,320 skull CT scans were available. After categorization by breed, total scans available per group varied from 13 to 310. There were insufficient scans available for giant size breeds of any skull type and extra small dolichocephalic. Thus, these categories were excluded from the evaluation. In total there were 176 skulls and 704 roots evaluated. There were 2–9 different breeds represented in each category with 1–15 scans per breed (Table 1). The mean facial index per skull type was 282.82 for brachycephalic, 134.27 for mesaticephalic, and 110.78 for dolichocephalic (Figure 2).

Lingual root location was the most common configuration representing 50% of all roots evaluated, and 64.2% of all dogs assessed had at least one root in the lingual position. Dorsal root location comprised 40.3% and buccal root location 9.7% of all roots evaluated (Tables 2, 3).

**Table 2:** Prevalence of buccal, dorsal, and lingual root location per skull type/size group. The most common conformation per group is in bold.

**Skull Type**	**Size**	**% Buccal Roots**	**% Dorsal Roots**	**% Lingual Roots**
Brachycephalic	Extra Small	23.4% (15/64)	3.1% (2/64)	73.4% (47/64)
	Small	43.8% (28/64)	7.8% (5/64)	48.4% (31/64)
	Medium	18.8% (12/64)	53.1% (34/64)	28.1% (18/64)
	Large	3.1% (2/64)	32.8% (21/64)	64.1% (41/64)
	All	22.3% (57/256)	24.2% (62/256)	53.5% (137/256)
Mesaticephalic	Extra Small	10.9% (7/64)	10.9% (7/64)	78.1% (50/64)
	Small	3.1% (2/64)	28.1% (18/64)	68.8% (44/64)
	Medium	0.0% (0/64)	50.0% (32/64)	50.0% (32/64)
	Large	0.0% (0/64)	64.1% (41/64)	35.9% (23/64)
	All	3.5% (9/256)	38.3% (98/256)	58.2% (149/256)
Dolichocephalic	Small	3.1% (2/64)	42.2% (27/64)	54.7% (35/64)
	Medium	0.0% (0/64)	70.3% (45/64)	29.7% (19/64)
	Large	0.0% (0/64)	81.3% (52/64)	18.8% (12/64)
	All	1.0% (2/192)	64.6% (124/192)	34.4% (66/192)
Normocephalic	Small	3.1% (4/128)	35.2% (45/128)	61.7% (79/128)
(M+D)	Medium	0.0% (0/128)	60.2% (77/128)	39.8% (51/128)
	Large	0.0% (0/128)	72.7% (93/128)	27.3% (35/128)
	All	1.0% (4/384)	56.0% (215/384)	43.0% (165/384)
All	Extra Small	17.2% (22/128)	7.0% (9/128)	75.8% (97/128)
	Small	16.7% (32/192)	26.0% (50/192)	57.3% (110/192)
	Medium	6.3% (12/192)	57.8% (111/192)	35.9% (69/192)
	Large	1.0% (2/192)	59.4% (114/192)	39.6% (76/192)
	All	9.7% (68/704)	40.3% (284/704)	50.0% (352/704)

**Table 3:** The percentage of patients with one or more of the indicated root locations out of the total number of patients evaluated for each group.

**Skull Type**	**Size**	**% Dogs with** >**1 Buccal Root**	**% Dogs with** >**1 Dorsal Root**	**% Dogs with** >**1 Lingual Root**
Brachycephalic	Extra Small	37.5% (6/16)	6.3% (1/16)	81.3% (13/16)
	Small	56.3% (9/16)	12.5% (2/16)	62.5% (10/16)
	Medium	37.5% (6/16)	75.0% (12/16)	37.5% (6/16)
	Large	6.3% (1/16)	56.3% (9/16)	87.5% (14/16)
	All	34.4% (22/64)	37.5% (24/64)	67.2% (43/64)
Mesaticephalic	Extra Small	31.3% (5/16)	31.3% (5/16)	100.0% (16/16)
	Small	6.3% (1/16)	37.5% (6/16)	81.3% (13/16)
	Medium	0.0% (0/16)	62.5% (10/16)	62.5% (10/16)
	Large	0.0% (0/16)	81.3% (13/16)	56.3% (9/16)
	All	9.4% (6/64)	53.1% (34/64)	75.0% (48/64)
Dolichocephalic	Small	6.3% (1/16)	75.0% (12/16)	81.3% (13/16)
	Medium	0.0% (0/16)	81.3% (13/16)	37.5% (6/16)
	Large	0.0% (0/16)	81.3% (13/16)	18.8% (3/16)
	All	2.1% (1/48)	79.2% (38/48)	45.8% (22/48)
Normocephalic	Small	6.3% (2/32)	56.3% (18/32)	81.3% (26/32)
(M+D)	Medium	0.0% (0/32)	71.9% (23/32)	50.0% (16/32)
	Large	0.0% (0/32)	81.3% (26/32)	37.5% (12/32)
	All	2.1% (2/96)	69.8% (67/96)	56.3% (54/96)
All	Extra Small	34.4% (11/32)	18.8% (6/32)	90.6% (29/32)
	Small	22.9% (11/48)	41.7% (20/48)	75.0% (36/48)
	Medium	12.5% (6/48)	72.9% (35/48)	45.8% (22/48)
	Large	2.1% (1/48)	72.9% (35/48)	54.2% (26/48)
	All	16.5% (29/176)	54.5% (96/176)	64.2% (113/176)

### Effect of skull type

**Figure 3:**
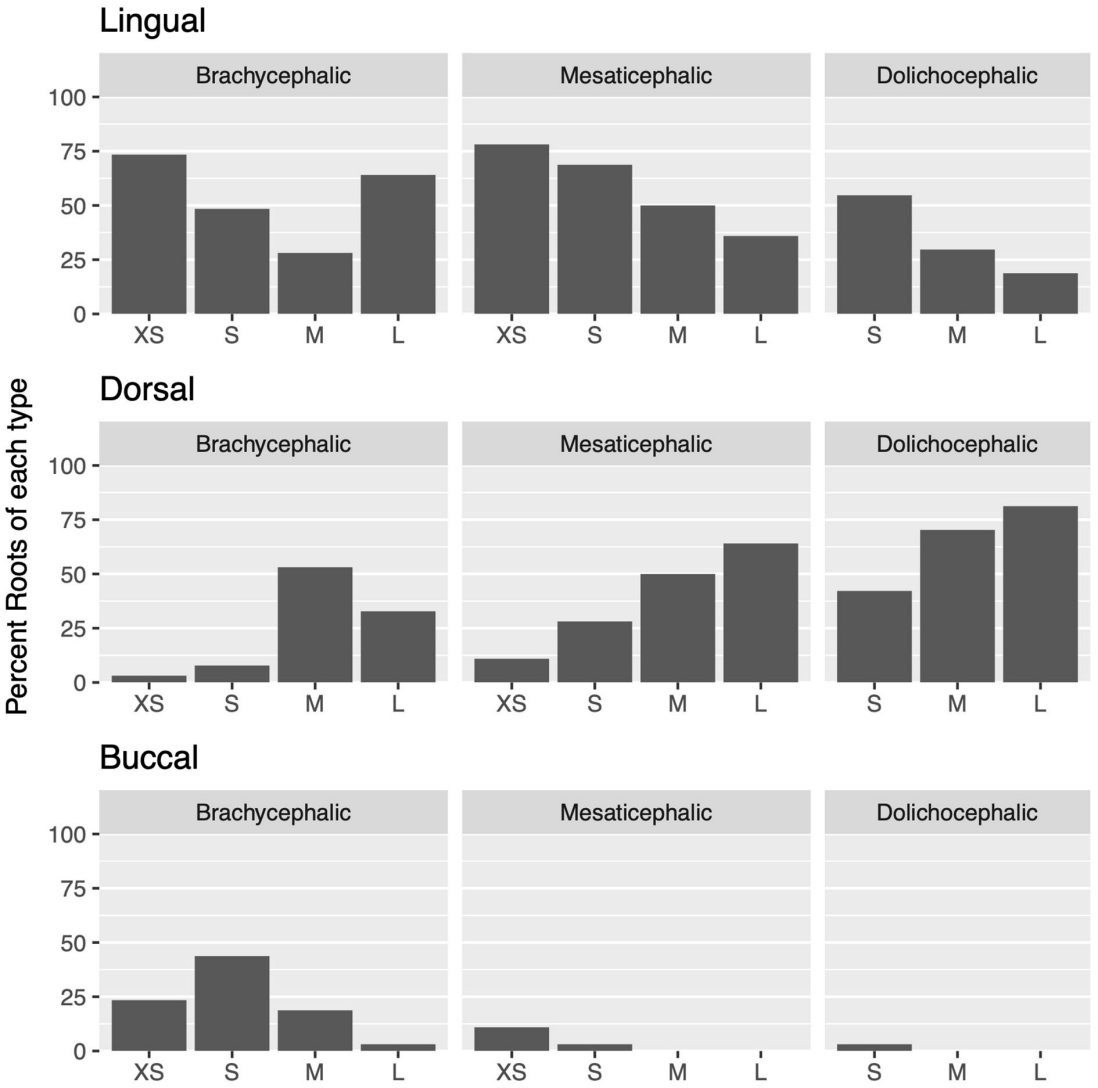
For each skull type and size group, the percent of each root type compared to the total number of roots is depicted.

**Figure 4:**
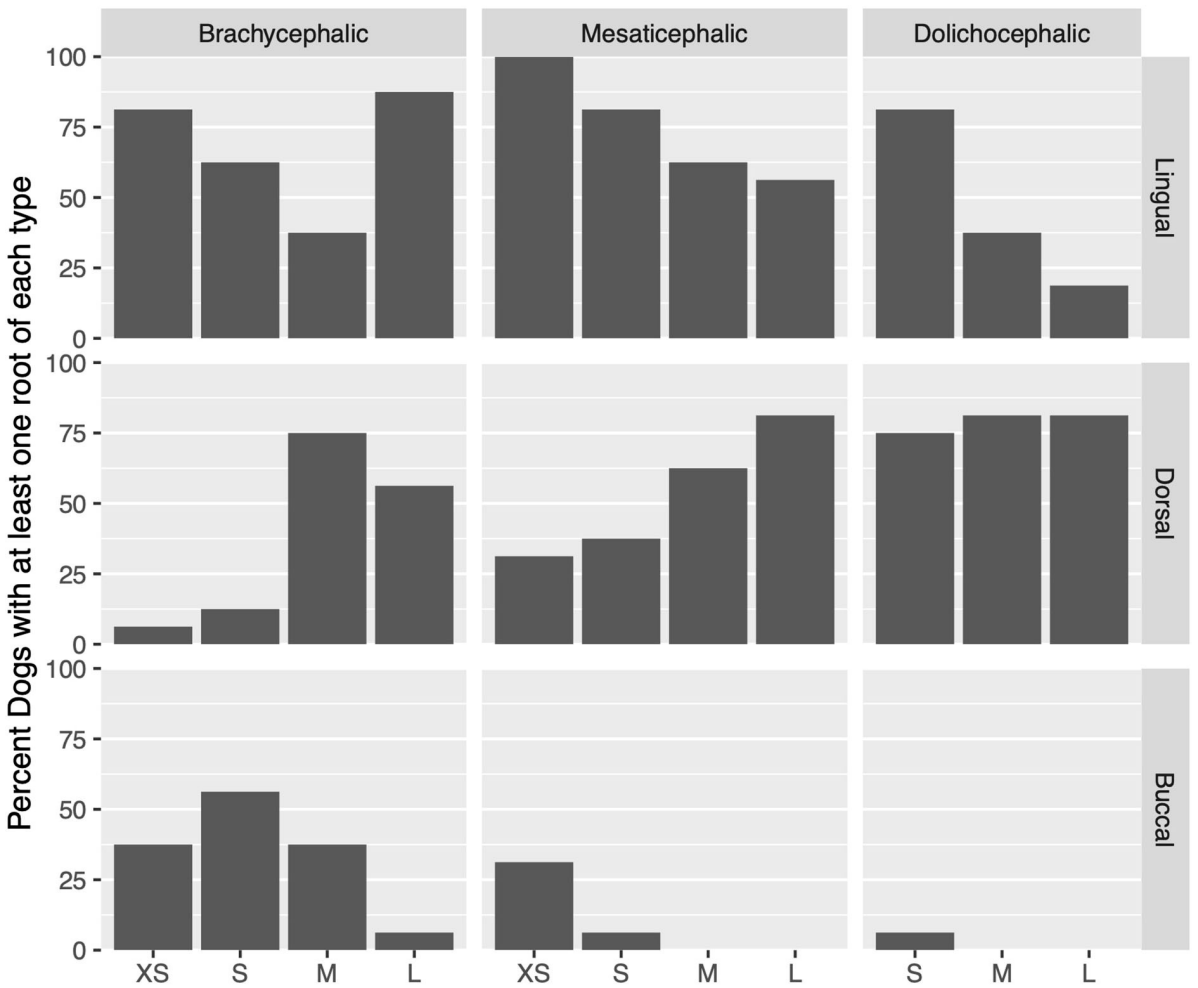
For each skull type and size group, the percent of dogs with at least 1 root in a localization (buccal, dorsal, or lingual) is represented.

Lingual root localization was the most common conformation in dogs with mesaticephalic (58.2% of roots) and brachycephalic (53.5% of roots) skull types (Figure 3). At least one root was in the lingual position in 75% of mesaticephalic dogs, 67.2% of brachycephalic dogs, and 45.8% of dolichocephalic dogs (Figure 4). Lingual roots were significantly more likely in mesaticephalic compared to dolichocephalic dogs (*p* = 0.040).

Buccal root location was relatively more common in brachycephalic patients, where 22.3% of roots were buccal, than in dolichocephalic or mesaticephalic patients, where only 2.5% of all roots were buccal. At least one root was buccally located in 34.4% of brachycephalic dogs. This was significantly more often than in dolichocephalic, mesaticephalic, and normocephalic dogs (*p* < 0.001).

Dorsal root location was the most common conformation in dolichocephalic dogs representing 64.6% of total roots and 79.2% of dolichocephalic patients having at least one root in this location. Dorsal roots were more common in dolichocephalic (*p* < 0.001), mesaticephalic (*p* = 0.018), and normocephalic (*p* < 0.001) dogs compared to brachycephalic dogs.

### Effect of size

Regardless of skull type, as size increased, the frequency of buccal and lingual roots decreased, and the frequency of dorsal roots increased (*p* < 0.001 for all root locations; Figure 3).

In extra small dogs, lingual root localization was most common (75.8% of roots) with 90.6% of dogs having at least one lingual root. There were no significant differences in root localization between extra small dogs with different skull types. Of note, 100% of mesaticephalic extra small dogs had at least 1 lingual root.

In small dogs, lingual root localization remained most common (57.3% of roots) with 75% of dogs having at least one lingual root. The likelihood of having at least 1 lingual root was less in brachycephalic (62.5%) compared to normocephalic (81.3%) small dogs. Buccal roots were relatively more common in brachycephalic (43.8%) than mesaticephalic (3.1%, *p* = 0.007) and dolichocephalic (3.1%, *p* = 0.007) small dogs. Conversely, dorsal root location was significantly more common in small dolichocephalic (42.2%, *p* = 0.002) and normocephalic dogs (35.2%, *p* = 0.006) compared to brachycephalic (7.8%) dogs.

In medium-sized dogs, the proportion of roots with dorsal root location increased (to 57.8%) compared with small dogs and became the most common configuration, compared to lingual (35.9%) and buccal (6.3%) positions, which both became less common. Only 45.8% of dogs were found to have at least 1 lingual root, and this was relatively more likely in mesaticephalic patients (62.5%) vs. others (37.5%). Buccal roots were significantly more likely in brachycephalic dogs compared to medium dolichocephalic, mesaticephalic, and normocephalic dogs (*p* = 0.030, 0.030, <0.001, respectively).

In large breed dogs, dorsal root location remained the most common configuration (59.4%) while buccal (1%) position became rare. More than half (54.2%) of large breed dogs still had at least one lingual root. Among large breed dogs, dorsal roots were significantly more likely in those with dolichocephalic (*p* = 0.002) and normocephalic (*p* = 0.001) skull types compared to large brachycephalic dogs. In fact, lingual root position was most common in brachycephalic patients (64.1%) compared to mesaticephalic (35.9%) and dolichocephalic (18.8%). The likelihood of encountering at least 1 lingual root in brachycephalic (87.5%) large dogs was also higher than in mesaticephalic (56.3%) and dolichocephalic (18.8%) patients.

### Straddle roots

Straddle roots were uncommon, appearing in 12 patients for a total of 20 straddle root teeth. Straddle roots were seen significantly more commonly in brachycephalic dogs regardless of size (*p* = 0.023), with 13 of the 20 observed straddle root teeth occurring in brachycephalic patients. In brachycephalic and mesaticephalic breeds, increasing size was associated with decreasing straddle root count (*p* = 0.040 and 0.034, respectively). Straddle roots were not observed in dolichocephalic patients.

### Mesial and distal root symmetry

Left-right asymmetry of the mesial roots was observed in 11 dogs (6.3%) and of the distal roots in 7 dogs (4.0%). Eight dogs (4.5%) had left-right asymmetry of both the mesial and distal roots. Thus, the vast majority of patients (85.2%) exhibited mesial and distal roots that were in the same position relative to the MC bilaterally. Root location asymmetry was not observed substantially more frequently in any particular size- or skull-type group.

## Discussion

The risk of iatrogenic complication during surgical extraction of the mandibular first molar is highest with the lingual root location. Specifically, if the excessive buccal bone is removed during the surgical approach the MC will be contacted with the high-speed drill before the root. Trauma to the structures within the canal could lead to severe hemorrhage or paresthesia of the neurovascular bundle (1–3, 8–10). Thus, the buccal or dorsal root location is favorable. Our study supported that in a diverse population of patients the lingual root location remains the most common conformation consistent with previous studies (1, 3). However, this location is relatively more common in extra small and small breed normocephalic dogs, and in large breed brachycephalic dogs—particularly boxers.

Previous studies evaluating the mandibular first molar root location in dogs found a relatively higher prevalence of lingual roots. Specifically, a study that evaluated dogs <15 kg with a variety of skull types (3), closely resembling the extra small and small categories of the present study, found lingual roots comprised 82.8% (341/412) of roots compared to 64.7% (207/320) in the present study. The same study found buccal to be the second most common (14.3%, 59/412), and dorsal least common (3.0%, 12/412). Comparatively, the present study found buccal (16.9%, 54/320) and dorsal (18.4%, 59/320) roots in similar frequency. These differences can most likely be attributed to breed differences within the two cohorts. It is surprising, however, that dorsal roots were so rare in the Chia study as almost 25% of their cohort was dolichocephalic (dachshunds). This skull type in our cohort had relatively more dorsal roots even in small breed dogs. This breed in particular may be less likely to have dorsal root confirmation compared to other dolichocephalic patients. Larger sample sizes comparing dachshunds to other dolichocephalic dog breeds would be required to confirm this.

A separate study on mesaticephalic dogs also found lingual roots to be more common (81.4%, 329/404 roots) compared to the present study (58.2% lingual root location in mesaticephalic patients). This is most likely attributed to the increased patient size within our cohort. Within the Berning study, 97.2% of dorsal roots were in dogs with a mandibular height >20 mm (1). Presuming that increasing mandibular height is associated with increasing patient size (11–13), the findings of both studies are in agreement that dorsal root frequency increases and lingual location decreases in mesaticephalic patients as size increases.

In fact, regardless of skull type, it was found that size was significantly associated with root location. The average lingual root count decreased as size increased; the frequency of lingual roots was highest in extra small patients assessed in this study, and second highest in small patients, supporting, in part, our initial hypothesis. From a clinical standpoint, what is more impactful than total root count is the likelihood that at least one root is in a lingual position. It was found that this decreased from 90.6% in extra small patients to 45.8% in medium patients. In extra small and small patients, especially those with a mesaticephalic skull type, extraction should be performed with a higher degree of caution than in a medium-large patient, and buccal bone should not be removed apical to the dorsal aspect of the MC as this will likely result in contact with the inferior alveolar neurovascular bundle.

Surprisingly, it was found that among small and medium patients, it was less common for dogs with a brachycephalic skull type to have lingual root localization, although this was not statistically significant. In fact, the majority of buccal roots observed in this study were seen in brachycephalic patients (83.8%), and across all sizes, brachycephalic subjects were more likely to have buccal roots (*p* < 0.001). This was least pronounced in the large brachycephalic category, which had only one subject with a buccal root, and instead was observed to have a high number of lingual roots compared to other large breed dogs. While some categories, like extra small and large mesaticephalic, consisted of 9 breeds in the final study cohort, other categories, like extra small brachycephalic and large dolichocephalic, consisted of more than half of a single breed. The large brachycephalic group consisted of the highest percentage of a single breed represented (93.8% or 15/16 boxers). This suggests that the boxer breed may have a greater association with lingual root location than other large dog breeds and additional caution should be employed when performing extractions in this breed in particular.

Although not as pronounced as the effect of brachycephalic skull type on root location, dolichocephalic patients also showed less lingual root localization. Within this skull type, dorsal root location was significantly more common, especially in larger patients. A notable limitation in the evaluation of this group, however, is that the facial index could not be validated against published values. Specifically, only 6/149 evaluated skull CTs from breeds that are commonly accepted as dolichocephalic fit into the defined facial index of <96. Using the commonly accepted dolichocephalic breeds, the interquartile range of the facial index measurements of all dolichocephalic dogs used in this study does not overlap with the interquartile range of the mesaticephalic dogs (Figure 2). This data suggest that the true reference values for dolichocephalic dog breeds may be higher than previously reported, and still distinguishable from mesaticephalic ranges. For this reason, we elected to still maintain the dolichocephalic group, rather than excluding this group altogether. In the future, it would be useful to investigate and establish revised reference ranges for each of the three skull types to better understand the categorizations of patients and predict the differences between them.

Straddle roots and left-to-right asymmetry of mesial roots and distal roots were rarely observed. This is consistent with previous findings (1, 3). Thus, regardless of skull conformation and breed size, there should be a minimal clinical concern for these configurations.

## Conclusions

This study was the first to evaluate the anatomical relationship between the mandibular first molar roots and mandibular canal in a diverse patient population. Weight is protective in dogs with normocephalic skull type with the risk of lingual root location decreasing with increasing size. In small and medium patients, lingual root localization is most common in dogs with mesaticephalic skull types. Extra caution should be employed during the removal of buccal alveolar bone for surgical extractions in extra small and small (≤ 13.6 kg) breed dogs and boxers as lingual tooth root location is most common in these cohorts. However, even in the lowest risk cohort (large dolichocephalic), at least 1 lingual root was seen in approximately 1 in every 5 dogs (18.8%), thus caution regardless of skull conformation is recommended.

## Data availability statement

The original contributions presented in the study are included in the article/supplementary material, further inquiries can be directed to the corresponding author.

## Author contributions

EG: project concept, study design, scan evaluation, manuscript writing, and graphic presentation. SG: project concept, study design, manuscript writing, and manuscript review. AR: statistical analysis and graphic presentation. All authors contributed to manuscript revision, read and approved the submitted version.

## Funding

This project was supported by a Morris Animal Foundation Veterinary Student Scholar Program Grant (Maf grant # D21CA-601).

## Conflict of interest

The authors declare that the research was conducted in the absence of any commercial or financial relationships that could be construed as a potential conflict of interest.

## Publisher's note

All claims expressed in this article are solely those of the authors and do not necessarily represent those of their affiliated organizations, or those of the publisher, the editors and the reviewers. Any product that may be evaluated in this article, or claim that may be made by its manufacturer, is not guaranteed or endorsed by the publisher.
